# Modern Sociology

**Published:** 1903-10-24

**Authors:** 


### Modern Sociology.

### THE LONDON SCHOOL BOARD AND PUBLIC HEALTH.

The School Board of London, if doomed to die, is determined to die as a British institution should—performing its duty to the last. It has just published a syllabus of the lectures on health given in its continuation schools during the current session. This has been drawn up by Dr. Robert Collie, Medical Superintendent of Hygiene, First Aid, and Home Nursing Classes, for the guidance of the doctors who are to give the lectures in the different schools. The syllabus is not meant as a hard and fast rule, and the lecturers are advised, if there is any part of the subject which they feel themselves especially well qualified to treat, or which is of particular importance to the district where they are lecturing, to give special attention to that. It is to be hoped that the first part of this permission will not be abused. Enthusiasts are not good popular lecturers, for they are apt to forget, in the first place, that their speciality does not cover the whole of the subject, and, in the second, that such an audience as these medical gentlemen will address in the Board schools has not the preliminary scientific knowledge to understand the meaning of details which are to themselves as simple as the alphabet. We remember a case in which an ardent bacteriologist devoted an entire lecture to the deeds and misdeeds of bacilli in such a fashion that his audience of working women imagined the smallest of these to be at least as large as pediculi—and would have been much more edified if he had taught them how to deal with the latter. But the advice to dwell with special emphasis on those questions of health which are of the greatest importance to the listeners in their persons and in their homes is wholly good, and it is to be hoped that the lecturers will bear it heartily in mind.

In itself the syllabus is thoroughly practical. Beginning with the house itself, the successive lectures treat of personal and domestic cleanliness, air, food, and water, while the two final lectures of the general course deal with the spread of infectious diseases and the means by which this may be prevented, and the prevention of communicable skin disease in school life. Besides this course of lectures, which is open to all, there are four special addresses for women only. These are devoted to the care of infants and children, to the causes of ill-health in woman, and to the various methods of cooking. Of course lectures alone often fail to convey much practical instruction to the comparatively illiterate—by which we mean not people who cannot sign their names, but those who have not the habit of grasping the relative importance of facts as they are presented to them. It will often be found, if one talks over a subject with such people after a lecture has been delivered upon it, that all they have remembered is a simile or an anecdote by which the lecturer illustrated a fact—the fact itself being quite forgotten. But what is seen is remembered. Therefore it is enjoined on the gentlemen who are to deliver these lectures that they are, so far as possible, to illustrate them by means of diagrams and experiments. For the structure of the body it is possible only to have diagrams and models, but many of the most practical details of health will be illustrated by suitable experiments. Thus the methods and merits of ventilation are to be shown both by models of ventilators—including one that can be easily fixed up by the householder himself—and by showing how the absence of air affects a lighted candle or match. The comparative inflammability of popular clothing fabrics can easily be shown in a practical manner, and experiments nearly as simple will show why hygienists are so strenuous about the purity of water and the sterilising of milk. At every lecture there will be something to impress upon the listeners the reasons for the care of health both by private individuals and by public authorities. It may be hoped that, as one result, the latter will find their path less beset with difficulties than is now the case.

Of course we know that those who need such instruction most will not go to the evening schools to profit by it. But that is no reason for not giving health lectures, nor for withholding from the School Board our appreciation of their efforts to teach the people the duty of sanitation. For besides those who err through indifference, there are those who err through ignorance alone, and who would do better if they knew. Let these be taught, and they will prove the best of health missioners. People are most easily influenced by those of their own class. The housewives who regard the sanitary officers as folk who talk cleanliness because they are paid to do so, and, when voluntary missionaries of a higher class than their own advise it, look upon the advice as simply a caprice of the rich, in no way applicable to the needs of working-class life, will follow, though only tentatively and shamefacedly, the sanitary and domestic improvements accepted by a neighbour of their own, who obviously has no larger income and no smaller a family than they have themselves. Thus the effect of these lectures may be very far-reaching even though the actual attendance may not be enormous. To many people free instruction of every kind seems to be waste, for they are convinced that those for whom it is intended will be indifferent to it, because they have not to pay for it. The dollar test is certainly a good one for other things than emotional charity, and free education which is also compulsory may not be appreciated. But the attendance at the evening schools is voluntary, it is paid for, and it implies, besides the trifling money payment, the sacrifice of the scanty leisure or the hardly-won amusement of those who come. Evening classes have to stand that severest test of all—the wishes of the beneficiaries. We hope that the attendance at these health lectures will show that among the poorer class of Londoners there are many who really want to learn those essentials of health-seeking and health-keeping which are of the utmost importance not only to themselves and their own families, but to all their neighbourhood, and ultimately to the city in which they live.

